# Neural Correlates of the DEEPP (Anti-suicidal Response to Ketamine in Treatment-Resistant Bipolar Depression) Study: Protocol for a Pilot, Open-Label Clinical Trial

**DOI:** 10.2196/41013

**Published:** 2023-01-27

**Authors:** Yuliya Knyahnytska, Reza Zomorrodi, Tyler Kaster, Daphne Voineskos, Alisson Trevizol, Daniel Blumberger

**Affiliations:** 1 Centre for Addiction and Mental Health Toronto, ON Canada

**Keywords:** bipolar depression, suicidality, ketamine intervention, neurophysiological markers of response

## Abstract

**Background:**

Suicide is among the top 10 leading causes of death worldwide. Of people who died by suicide, the majority are diagnosed with depression. It is estimated that 25%-60% of people with bipolar depression (BD) will attempt suicide at least once, and 10%-15% will die by suicide. Several treatments, such as lithium, clozapine, electroconvulsive therapy, and cognitive behavioral therapy, have been shown to be effective in treating suicidality. However, these treatments can be difficult to tolerate or may take months to take effect. Ketamine, a glutamate N-methyl-D-aspartate antagonist, has been shown to have rapid antisuicidal effect and antidepressant qualities, and is thus a promising intervention to target acute suicidality in patients with BD. However, the biological mechanism underlying its therapeutic action remains poorly understood. Enhancing our understanding of underlying mechanisms of action for ketamine’s effectiveness in reducing suicidality is critical to establishing biological markers of treatment response and developing tailored, personalized interventions for patients with BD.

**Objective:**

This is an open-label clinical trial to test the safety and feasibility of repeated ketamine infusions to treat acute suicidality. The primary objective is to test the safety and feasibility of ketamine intervention. The secondary objective is to examine ketamine’s potential neurophysiological mechanisms of action by assessing cortical excitation and inhibition to determine potential biomarkers of clinical response. Other objectives are to evaluate the effect of ketamine on acute suicidality and other clinical outcomes, such as depressive symptoms and quality of life, to inform a future larger trial.

**Methods:**

This open-label clinical trial aims to test the safety and feasibility of repeated ketamine infusions in patients with BD for suicidality and to assess ketamine’s neurophysiological effects. A sterile form of racemic ketamine hydrochloride will be administered over a 40-minute intravenous infusion 2 times per week on nonconsecutive days for 4 weeks (8 sessions). We will recruit 30 adults (24-65 year olds) over 2 years from an academic psychiatric hospital in Toronto, Canada.

**Results:**

This study is currently ongoing and actively recruiting participants. So far, 5 participants have completed the trial, 1 is currently in active treatment, and 8 participants are on the waitlist to be screened. We anticipate initial results being available in the fall of 2023. This proposal was presented as a poster presentation at the Research to Reality Global Summit on Psychedelic-Assisted Therapies and Medicine, held in May 2022 in Toronto, Canada.

**Conclusions:**

Developing effective interventions for acute suicidality in high-risk populations such as those with BD remains a major therapeutic challenge. Ketamine is a promising treatment due to its rapid antidepressant and antisuicidal effects, but its underlying neurophysiological mechanisms of action remain unknown.

**Trial Registration:**

ClinicalTrials.gov NCT05177146; https://clinicaltrials.gov/ct2/show/NCT05177146

**International Registered Report Identifier (IRRID):**

DERR1-10.2196/41013

## Introduction

### Background and Rationale

Suicide is among the top 10 leading causes of death, with progressively increasing rates and is a critical public health issue [1]. Bipolar depression (BD) is a serious psychiatric condition with a high mortality rate due to suicide [2]. Between 25% and 60% of those with BD will attempt suicide at least once, and 10%-15% will die by suicide [3-5]. Several interventions, such as lithium, clozapine, electroconvulsive therapy, and cognitive behavioral therapy, have demonstrated antisuicidal properties [4,5]. However, as polypharmacy is common in patients with bipolar illness [6], adding another treatment can be difficult to tolerate, or new treatments may take months to take effect. Therefore, there is an urgent need to develop robust interventions to treat suicidality in patients with BD rapidly. In addition, establishing biological markers of treatment response is critical in developing personalized, tailored treatment interventions.

A paradigm shift from a monoamine hypothesis to a plasticity-based hypothesis of depression represents a substantial advancement, where accumulating evidence suggests that glutamatergic or excitatory neurotransmission system dysfunction plays a pivotal role in depression [7-9]. There are 3 classes of glutamate-gated ion channels such as α-amino-3-hydroxy-5-methyl-4-isoxazole propionic acid, and kainite and *N*-methyl-d-aspartate (NMDA) receptors that transduce the postsynaptic signal [7]. NMDA receptors are distributed throughout the brain and are fundamental to excitatory neurotransmission and the normal functioning of the central nervous system (CNS) [7,8]. Ketamine is the first clinically used NMDA receptor antagonist with a primary mechanism of action closely linked to the blockade of the NMDA receptor at the phencyclidine site, which mediates excitatory synaptic transmission through the CNS [8-10].

Developing effective interventions for acute suicidality in high-risk populations, such as BD, remains a major therapeutic challenge. Ketamine is a promising treatment modality due to its rapid antisuicidal [11,12] and antidepressant [13] effects, with numerous studies demonstrating its safety and efficacy in subanesthetic doses administered over 40-minute intravenous (IV) infusions [11-13]. Despite growing interest in ketamine interventions in depressive disorders, our understanding of ketamine’s underlying neurophysiological mechanisms of action, especially concerning suicidality, remains very limited.

Noninvasive brain stimulation neurophysiological tools, such as transcranial magnetic stimulation (TMS), offer an opportunity to study human brain cortical physiology mechanisms in vivo with high temporal resolution [14]. The combination of TMS with a CNS pharmacological agent, such as ketamine, will provide a platform to explore the neurophysiological basis of the impact of ketamine on brain physiology, an important piece of work toward identifying neurobiomarkers of treatment response. Until present, only some studies have attempted to examine the excitatory and inhibitory circuits by using a range of TMS protocols during infusion of incremental doses of ketamine [15-17], primarily using very low ketamine doses or single doses in healthy volunteers. Currently, the prediction of the therapeutic response in those with mood disorders as measured by changes in cortical excitability continues to be in very early stages and has not been systematically tested, making this proposal uniquely positioned among other neurophysiological studies on ketamine and TMS. Our group has previously demonstrated impaired GABA (gamma-aminobutyric acid) inhibition from the motor cortex in patients with depression, a finding that was most pronounced in patients with treatment-resistant depression [16,18,19]. In addition, Croarkin et al [19] demonstrated impaired NMDA receptor–mediated excitation in depression.

### Objectives

The primary objective of this open-label pilot clinical trial is to examine the safety and feasibility of repeated subanesthetic doses (0.5-0.8 mg/kg) of ketamine administered over 40-minute IV infusions in patients with BD to treat acute suicidality. The secondary objective is to analyze the potential impact of ketamine on neurophysiological markers associated with cortical excitation and inhibition as a proxy of NMDA receptor activity in the cortex. Our scientific group has successfully applied TMS-electromyography (EMG) and TMS-electroencephalogram (TMS-EEG) paradigms to assess cortical activities in patients with psychiatric issues [15,18]. Thus, we will use these paradigms to investigate the impact of ketamine on cortical activities in patients with BD and suicidality. We have previously reported that intracortical facilitation is abnormal in depression [14,18] and is closely associated with NMDA receptor–mediated neurotransmission [9,18]. This study will evaluate cortical excitation and inhibition pre- and postketamine course in patients with BD and suicidality to determine a biological target of ketamine treatment response. We will use a TMS-EMG paradigm to investigate the impact of ketamine on cortical excitation via intracortical facilitation and cortical inhibition via short-interval cortical inhibition paradigms [19-21]. Next, we will compare the effect of ketamine on resting-state cortical oscillation via EEG. These results will provide important evidence for the role of NMDA receptors in cortical physiology, which may serve as a ketamine biomarker and would be a crucial breakthrough in determining potential predictors of clinical response for suicidality in patients with BD.

### Trial Design

The DEEPP (Anti-suicidal Response to Ketamine in Treatment-Resistant Bipolar Depression) study is designed as an open-label pilot clinical trial to assess (1) the feasibility of a series of IV ketamine infusions to treat suicidality in patients with BD and (2) ketamine’s neurophysiological markers associated with cortical excitation and inhibition.

## Methods

### Study Setting

Potential participants will be recruited from 1 site, specifically the Centre for Addictions and Mental Health, a large academic center located in Toronto, Ontario, Canada, over 2 years.

### Eligibility Criteria

*Inclusion criteria* are patients who (1) meet criteria for bipolar disorder according to DSM-V (Diagnostic and Statistical Manual of Mental Disorders, 5th edition) criteria as confirmed by the Mini-International Neuropsychiatric Interview (MINI) [22]; (2) meet criteria for a current depressive episode, confirmed with the 24-item Hamilton Rating Scale for Depression (HRSD-24) score of 14 and above [23]; (3) experiencing suicidal ideation as defined by a score of 9 or higher on the Scale for Suicide Ideation (SSI) [24,25]; (4) capable of providing consent; (5) outpatients; (6) able to speak and understand English; and (7) aged 24-65 years, inclusive.

*Exclusion criteria* are patients who (1) have a history of a substance use disorder within the past month or a lifetime history of ketamine substance use disorder, as confirmed by the MINI; (2) concomitant major unstable medical illness (eg, poorly controlled hypertension, enlarged prostate, renal- or urinary-related issues, the presence of cardiac decompensation/heart failure, results of liver function tests [alanine transaminase and aspartate transaminase] are 3 times or greater than the upper limit of normal readings); (3) pregnancy or the intention to become pregnant or breastfeeding during the study as confirmed by self-report. Female participants of reproductive potential must be willing to use a medically acceptable method of birth control which includes highly effective (eg, approved hormonal contraceptives, intrauterine device, tubal ligation) or double barrier (eg, male condom with a diaphragm, male condom with cervical cap) methods of contraception or abstinence if that is the usual and preferred lifestyle of the participant; (4) DSM-V diagnosis of any primary psychotic disorder, obsessive-compulsive disorder, posttraumatic stress disorder (current), unipolar depression, other subtypes of depressive disorders, or current psychotic symptoms as confirmed by the MINI; (5) current episode meeting criteria for mania/hypomania or mixed episode as per DSM-V criteria on the MINI or as determined by the study team; (6) primary diagnosis of severe personality disorder as assessed during the initial consultation with a study psychiatrist prior to study entry; (7) any significant neurological disorder (eg, a space occupying brain lesion, a history of stroke, a cerebral aneurysm, a seizure disorder, Parkinson disease, Huntington chorea, multiple sclerosis) as assessed through medical history review during the initial consultation with a study physician prior to study entry; (8) requiring a benzodiazepine with a dose equivalent to lorazepam 2 mg/day or higher; (9) being on any anticonvulsant (eg, lamotrigine, topiramate, carbamazepine) or opioid medication due to the potential of these medications to limit the efficacy of ketamine; (10) cognitive or physical impairment that may potentially interfere with IV ketamine administration or participants’ ability to stay in the same place for a 2-hour monitoring supervision as assessed through medical history review during the initial consultation with a study physician prior to study entry; (11) any intracranial implant (eg, aneurysm clips, shunts, cochlear implants) or any other metal object within or near the head, excluding the mouth, that cannot be safely removed given that we will be using TMS-EMG/EEG; (12) inability to secure escort to accompany them back home after ketamine sessions; or (13) any known allergy to ketamine or any component or ingredient of the ketamine preparation.

### Discontinuation Criteria

Participants will be discontinued from the study if they cannot safely continue the study based on any of the following criteria: (1) experience clinically significant worsening of depressive symptoms (50% increase in HRSD-24 scores from baseline on 2 consecutive ratings); (2) require in-patient hospitalization due to the presence of clinically significant suicidal ideation with imminent intent or attempted suicide; (3) develop clinically significant worsening of mood or physical symptoms (assessed by a study physician); (4) miss over 2 consecutive treatments during the study without the cause; (5) develop any medical illness that may be unstable; (6) experience a seizure; (7) become pregnant, or (8) withdraw consent.

### Intervention

#### Drug Characteristics, Distribution, and Storing

A sterile form of racemic ketamine hydrochloride (DIN: 02246795, 02246796) will be dispensed through the Centre for Addiction and Mental Health (CAMH) pharmacy. Ketamine will be administered intravenously over a 40-minute infusion using a Baxter FA-2021-056 pump. The CAMH research pharmacy will order the investigational product from the supplier (Sandoz Canada Inc) on behalf of the research team with the study investigator’s authorization and will be responsible for the receipt and responsible destruction of the investigational product. As per the manufacturer’s recommendation, the investigational product will be stored between 15°C and 30°C, protected from light and heat, and discarded within 28 days of initial use. Only dedicated medical personnel will have access to the investigational product and pick it up from the locked and password-protected storage on the day of the treatment. All treatment sessions will be delivered at CAMH’s Temerty Centre for Therapeutic Brain Intervention, a dedicated clinic providing electroconvulsive therapy to patients and thus equipped with a highly trained medical team.

#### Drug Administration and Scheduling

Ketamine will be administered 2 times per week for 4 weeks (a total of 8 treatment sessions). The dosing schedule will be determined based on the patient’s weight, clinical response, and tolerability (Table 1). The first treatment session dose will be calculated based on 0.5 mg/kg. Doses in this trial will range between 0.5 and 0.8 mg/kg.

**Table 1:** Intervention schedule.

Week	Session, intervention, and dosing
1	Start-up (first) dose to assess tolerability; 0.5 mg/kg intravenous infusion over 40 minutes
	Second infusion session (dose adjustment, if needed, 0.5-0.8 mg/kg)
2	Third infusion session (40 minutes; dose adjustment, if needed, 0.5-0.8 mg/kg)
	Forth infusion session (40 minutes; dose adjustment, if needed, 0.5-0.8 mg/kg)
3	Fifth infusion session (40 minutes; dose adjustment, if needed, 0.5-0.8 mg/kg)
	Sixth infusion session (40 minutes; dose adjustment, if needed, 0.5-0.8 mg/kg)
4	Seventh infusion session (40 minutes; dose adjustment, if needed, 0.5-0.8 mg/kg)
	Eighth infusion session (40 minutes; dose adjustment, if needed, 0.5-0.8 mg/kg)

#### Dose Adjustment

If the first session is tolerated well and the patient has no side effects, the dose can be further increased up to (but not exceeding) 0.8 mg/kg. Participants will have a weekly follow-up with the study’s physician. The study physician will determine the exact doses on a case-by-case basis based on treatment tolerability, safety, and clinical response. From principal investigator’s (PI) previous clinical experience treating patients with ketamine, dose adjustment occurs within the first 1-3 sessions, and patients continue with stable doses following sessions 2 and 3. However, dose adjustment can also occur after session 3 if the participant experiences side effects with repeated infusions. The procedure around dose adjustment is a part of this trial’s feasibility; this information will be collected by the study physician during weekly follow-ups and incorporated into the research protocol for further studies in the future.

#### Monitoring Schedule

All patients will stay on-site for the administration and monitoring period, calculated as 2 hours from the start of IV infusion per consensus guidelines in ketamine administration. Patients will stay in a separate private space and be provided with noise-cancellation headphones and an eye mask to minimize environmental disruptions. Patients will be closely monitored by available trained personnel, with a medical doctor present on-site for the entire supervision period. Specifically, IV induction will be performed under the direct supervision of an anesthesiologist or delegate and psychiatrist, who will remain present on-site for the entire duration of the 40-minute ketamine infusion. Trained personnel are available on-site to closely monitor the postinfusion period, where vital signs (ie, heart rate, blood pressure, oxygen saturation level) will be monitored every 30 minutes. Each patient will be closely monitored for the entire IV infusion duration and the postinfusion period for at least 2 hours. The overall time per treatment session is at least 2 hours starting from the time of infusion initiation. This includes the 40-minute infusion period and 80 minutes for postinfusion monitoring. Any adverse events will be managed by the medical team administering the treatment. If required, appropriate medications will be provided to manage treatment-related side effects and any adverse events as defined by clinical site policies and regulations. Clinical and neurophysiological assessments will be administered according to the trained research personnel’s schedule for study visits. There is a total of 16 study visits in this trial, which includes treatment sessions and follow-ups.

#### Potential Risks and Mitigation Strategies

Given the potential risks described below, trained medical personnel will be present during the administration and for the entire duration of the 2-hour monitoring period.

Ketamine is classified as a schedule I controlled substance due to its potential for abuse and addiction and can be abused in a number of ways, including via injection, snorting, or orally. Ketamine can produce vivid dreams and a feeling that the mind is separated from the body, referred to as “dissociation.” Regular users of ketamine can become tolerant to the dissociative effects of the drug, meaning an increasing amount is needed to achieve the same effect. Drug-related risks include (1) psychiatric symptoms such as fatigue, dizziness, anxiety, visual and auditory disturbances, panic attacks, increased irritability, and changes in mood and behavior; (2) medical symptoms such as transient increases in blood pressure and heart rate, an increase in the need to urinate, headaches, vision changes, chest pain, shortness of breath, confusion, memory impairment, anaphylaxis; and (3) a rare risk of dependency. To address these risks, in this trial, ketamine will be dispensed by the research pharmacy and administered by a trained medical professional, and the patient will receive close supervision and monitoring. Doses are individually calculated, and treatment sessions are structured to prevent tolerance building. Patients will be strongly discouraged from using ketamine outside of the context of this trial and will be informed that they can be discussed with the study physician if they have questions.

IV route–associated risks are infiltration, hematoma, air embolism, phlebitis, extravascular drug administration, and intraarterial injection. Most of these complications are transient and resolve with conservative monitoring. An intraarterial injection is rare but as threatening. An anesthesiologist is present on-site for the IV infusion and will attend if needed, per site rules and regulations.

#### Clinical Assessment Risks

Answering multiple questions at times can be distressing. These adverse reactions are mostly brief, transient, and rarely have long-term implications.

#### TMS-EMG/EEG Risks

Single-pulse TMS is now in routine clinical diagnostic use in hundreds of neurophysiological laboratories worldwide. The ability of TMS to noninvasively stimulate brain areas presents a significant advance beyond techniques that require the invasive method of direct cortical or transcranial electrical stimulation. Magnetic fields pass through the scalp and skull without the impedance encountered by direct electrical stimulation, permitting enhanced control over the site and intensity of stimulation. The induced electrical current is well below that which is expected to cause harm to nervous tissue; thus, stimulation at <1 Hz carries virtually no risk of seizure and is therefore classified as a nonsignificant risk device. In numerous studies, single-pulse TMS has been found to pose no significant health risk to properly screened healthy volunteers.

### Outcomes

The primary outcome of this pilot trial is to examine the safety and feasibility of repeated subanesthetic doses (0.5-0.8 mg/kg) of ketamine administered over 40-minute IV infusions in patients with BD to treat acute suicidality based on recruitment numbers, attrition rate, and a number of adverse events in the trial. The secondary outcome is to analyze the potential impact of ketamine on neurophysiological markers associated with cortical excitation and inhibition as a proxy of NMDA receptor activity in the cortex. The tertiary outcomes are to assess the intervention’s potential effect on clinical outcomes, such as acute suicidality, depressive symptoms, and quality of life.

### Participant Timeline

**Figure 1:**
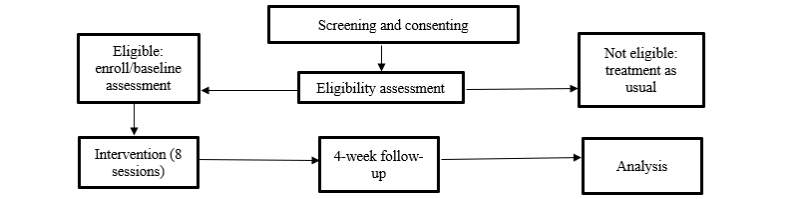
Flowchart of the study design.

We intend to recruit 30 participants, adults 24-65 years old, diagnosed with BD and experiencing suicidal thoughts in their current episode, over the duration of 2 years from 1 site (CAMH). The trial period is shown in the CONSORT (Consolidated Standards of Reporting Trials) diagram (Figure 1).

### Sample Size

We calculated our sample size based on this study design, which is to test the safety and feasibility of ketamine infusions. We calculated that a minimum sample size of 30 participants is needed for the proposed investigation.

### Recruitment

This is a single-site open-label pilot safety and feasibility study that will be conducted at the CAMH. The CAMH is a large urban academic institution offering psychiatric care to a diverse population across the province. We intend to recruit participants through CAMH General Division Ambulatory services, which annually provides over 500 consultations for mood-related concerns. The study’s PI is a staff psychiatrist and clinician-scientist in General Division who provides outpatient services to a large population with mood disorders at local and provincial levels. PI is also an interventional psychiatrist working as a part of the Therapeutic Brain Intervention Centre, seeing many patients with treatment-resistant and severe mood disorders. She is also US certified in ketamine interventions and a member of the American Society for Ketamine Physicians (ASKP). This study group has extensive experience with recruiting patients for clinical trials as the PI of this study and coinvestigators are clinician-scientists who provide care to a large number of patients with mood disorders within the context of clinical trials. Thus, we believe recruiting 30 participants over 2 years is feasible.

In addition to group expertise, we will utilize a CAMH-wide recruitment strategy, specifically the Clinical Engagement and Research Recruitment (CLEARR) model, where all new referrals to the CAMH clinics are prescreened, and all patients are informed about clinical research at the time of their initial clinical assessment. The study site will make efforts to recruit a representative and ethnically diverse sample. CAMH services a very diverse inner-city population through numerous satellite clinics in several minority neighborhoods throughout the city. Toronto has diverse cultural representation as almost 75% of its population, aged 15 and older, have direct ties to immigration. In this study, we anticipate our recruitment will follow the ethnic distribution of White/non-Hispanic 50%, Asian 20%, Southeast Asian 15%, Black/non-Hispanic 10%, and Hispanic 5%. We also expect that 60% of our population enrolled at the CAMH site will be female based on our prior experience with clinical trials at the CAMH.

### Data Collection Methods

Data will be collected on a baseline assessment, weekly during the course of IV ketamine, within a week after the completion of the intervention, and following 4 weeks for a final follow-up. Assessments will include psychiatric assessment, physical examination, and clinical assessments through manualized validated clinical scales (Multimedia Appendices 1 and 2).

*Psychiatric assessment* will include a consultation with a study physician to confirm a diagnosis. Demographic information, medical and psychiatric history, and concomitant medications will also be included.

A *physical examination* will include a physical exam performed by a study physician or a delegate, and blood work and electrocardiography for medical clearance according to standard clinical procedures also be reviewed by the study physician. Blood tests will include complete blood count with differential tests (white blood cells, red blood cells, hemoglobin, hematocrit, mean corpuscular volume, mean corpuscular hemoglobin, mean corpuscular hemoglobin concentration, red cell distribution width, mean platelet volume, platelet, nucleated red blood cell count, neutrophil count, lymphocyte count, monocyte count, eosinophil count, basophil count); blood chemistry test, including liver function (alanine transaminase and aspartate transaminase), kidney function (blood urea nitrogen and creatinine), and electrolytes (sodium, potassium, chloride, and phosphate); and thyroid function (thyroid-stimulating hormone). Study participants’ blood pressure will also be monitored before, during, and after treatment.

*Clinical assessments* will include (1) the MINI to clarify psychiatric inclusion and exclusion criteria; (2) the Antidepressant Treatment History Form (ATHF) to clarify on inclusion and exclusion criteria [26]; (3) the SSI will be administered at screening, baseline, weekly, and after treatment to confirm eligibility and to assess changes as a secondary outcome measure; (4) the HRSD-24, which will assess changes in depressive symptoms and will be administered at the baseline and after treatment; (5) the Young Mania Rating Scale (YMRS), which will be used to evaluate mania/hypomania symptoms at screening and throughout the study for eligibility and safety monitoring purposes [27]; and (6) World Health Organization Disability Assessment Schedule (WHODAS 2.0), which will be used as a standard measure of disability promoted and used at baseline and after treatment to assess changes in individual level of functioning [28].

*Neurophysiological assessments* will include (1) the Transcranial Magnetic Stimulation Adult Safety Screen to assess potential TMS risk factors; and (2) TMS-EMG/EEG. Our neurophysiological measures have been established and have a high test-retest reliability (ie, intraclass correlation >0.9) [18,19]. Data analysis will be performed using semiautomated methods developed and validated by our group [29]. TMS pulses will be administered to the left dorsolateral prefrontal cortex using a 7-cm figure-of-eight coil and 2 Magstim 200 stimulators (Magstim Company Ltd) connected via a BiStim module, and motor-evoked potential data will be collected using commercially available software (Signal; Cambridge Electronics). Each TMS session will include the establishment of the individual threshold for stimulation of motor cortex, and dorsolateral prefrontal cortex localization will be performed according to previously published methods [29,30]. The resting motor threshold will be determined by applying single pulses of TMS to the motor cortex while the coil is placed at the optimal position to elicit motor-evoked potentials from the right abductor pollicis brevis muscle. The resting motor threshold is defined as the minimum stimulus intensity that elicited a motor-evoked potential of >50 μV in more than 5 of the 10 trials [30].

TMS-EMG will be recorded from the abductor pollicis brevis with Ag-AgCl electrodes placed over the belly of the muscle. The signal will be amplified (Model 2024F; Intronix Technologies Corporation), filtered (band-pass 2 Hz-5 kHz), digitized at 5 kHz (Micro 1401; Cambridge Electronics Design), and stored in a laboratory computer for offline analysis. The participants will be instructed to relax throughout the study. Trials contaminated with voluntary muscle activity will be discarded.

### TMS-EEG

EEG will be used to evaluate TMS-induced cortical evoked activity. EEG recordings will be acquired through a 64-channel EEG system [30]. A 64-channel EEG cap will be used to record the cortical signal, and 4 electrodes will be placed on the outer side of each eye and above and below the left eye to closely monitor the eye movement artifact. All electrodes will be referenced to an electrode placed on the vertex positioned posterior to the Cz (midline central) electrode. Direct current EEG signals will be recorded with a 20-kHz sampling rate and with a low-pass filter of 300 Hz, which in pilot experiments was shown to avoid saturation of amplifiers and minimize the TMS-related artifact. The EEG data will be downsampled to a 1-kHz sampling frequency and segmented concerning the TMS stimulus such that each epoch includes a 1000-ms prestimulus baseline and a 1000-ms poststimulus activity. Epochs will be baseline corrected concerning the TMS-free prestimulus interval. The baseline-corrected poststimulus intervals (approximately 25-1000 ms) that are not contaminated by TMS artifact will be extracted and digitally filtered using a zero-phase shift 1-100-Hz band-pass filter (48 dB per octave). Records will be manually reviewed at this stage, and trials contaminated with muscle activity, movement, and TMS artifacts will be excluded from further analysis. Finally, the average signals at each recording site will be computed from the movement-free epochs (approximately 80 trials per participant) and fed into an automated eye-blink correction algorithm [30]. The eye-blink–corrected average EEG waveforms will be imported into MATLAB (The MathWorks Inc), and further analyses will be carried out using the EEGLAB toolbox [31]. Furthermore, methods in this approach will be conducted according to previously published combined TMS-EEG studies [29-31].

### Data Management

The REDCap (Research Electronic Data Capture) software will be used for data collection and overall study data management throughout this project. REDCap is a secure, web-based clinical data management and electronic data capture system and database. It is built and distributed by Vanderbilt University, with a local instance installed on a central CAMH server overseen by the CAMH REDCap Operations Committee. All data are stored on-site and backed up daily. The system is developed and managed in compliance with HIPAA (Health Insurance Portability and Accountability Act), PIPEDA (Personal Information Protection and Electronic Documents Act), and FDA21 CFR (US Food and Drug Administration Code of Federal Regulations Title 21) Part 11 regulations, providing functions such as defined user roles and privileges, user authentication and encryption, electronic signatures, deidentification of protected health information, comprehensive auditing features to record and monitor access and data changes, and a validated software development life cycle. This system will collect data, design electronic case report forms, data entry, monitor and clean, and query and export data sets for statistical analysis.

### Statistical Methods

Baseline participant characteristics will be reported and described using summary statistics—mean and SD for continuous data and number and proportion for categorical data. As the primary outcome is to assess feasibility, we will report on (1) recruitment numbers and attrition rates; (2) the number of individuals who experience side effects, and (3) the overall number and type of side effects. For neurophysiology, we will use an independent *t* test (paired 1-tailed) to compare components of TMS-evoked responses in the left dorsolateral prefrontal cortex in TMS-EEG data. In addition, we will apply a nonparametric cluster-based permutation test to investigate any significant changes in overall EEG channels. We will conduct a paired 1-tailed *t* test of the SSI and HRSD-24 scores from baseline to week 4 and monthly follow-up to assess preliminary clinical outcomes. We will also report the standardized mean difference as a measure of effect size (small: 0.2; medium: 0.5; large: 0.8).

### Monitoring

#### Trial Monitoring

We will perform internal quality management of study conduct, data collection, documentation, and completion through proactive study monitoring using our team-based monitoring approach. Monitoring will be conducted prior to, during, and after the study concludes to ensure that good clinical practice is maintained. All data monitoring, auditing, and harms reporting will be performed according to CAMH Research Ethics Board and regulatory standards. The study investigator and delegated research personnel will monitor study participants according to the study protocol and continually assess the patient for adverse outcomes; all adverse events will be assessed in real-time to determine if they meet the criteria for a “serious adverse event” and how the event was “related” to the study intervention.

#### Harms

Adverse events occurring as of the first administered dose of the investigational product and 30 days after the last administered dose will be collected. All reported adverse events will be followed up to the resolution, or an appropriate posttrial follow-up plan will be determined. Serious unexpected adverse drug reactions will be reported as required by Health Canada, the Food and Drug Regulations—Division 5. For this pilot trial, all relevant adverse events and all serious adverse events will be reported to the Research Ethics Board directly if they meet applicable reporting requirements.

#### Auditing

At the time of this publication submission, the study also passed the CAMH Quality Assurance internal auditing process. Quality control procedures will be implemented beginning with the data entry system, and data quality control checks that will be run on the database will be generated. Any missing data or data anomalies will be communicated for clarification/resolution.

### Research Ethic Approval

This study has received CAMH Research Ethics Board (approval number 179-2020) and Health Canada (approval number NOL251577) approvals.

### Consent

We will obtain informed consent from each participant who agrees to participate in the trial. Patients meeting the criteria will be referred to the research analyst/study coordinator to discuss the study purpose, procedures, potential risks, and rights as research participants. Consent forms describing the study intervention, study procedures, and risks will be given to each participant, and written documentation of informed consent will be required prior to completing the initial screening visit and starting the study intervention. Once consent is obtained, the research personnel will confirm that inclusion and exclusion criteria are met before proceeding with baseline testing. Patients will be informed that they can withdraw participation at any point during the study. The participants will be given a copy of the information and consent documents for their records. The informed consent process will be documented, and the form will be signed before the participant undergoes any study-specific procedures. The rights and welfare of the participants will be protected by emphasizing that the quality of their medical care will not be adversely affected if they decline to participate in this study (Multimedia Appendix 3).

### Confidentiality

Data confidentiality and confidentiality of the identities of the individuals participating in this study will be strictly maintained. Data forms that include identifying information will be kept in locked cabinets. Only the unique ID number assigned by the research coordinator will be used to represent participants during data entry, data transfer, data analysis, or other file management procedures. All information linking their identity will be kept separate from the research records. All information entered into a computer will be stored in a password-protected and encrypted file format on a secure server. If any participant withdraws from the study, any research information recorded for or resulting from the participant prior to the date that the participant formally withdrew consent will continue to be stored and used (in the manner described above) for research purposes (and will be disclosed by the investigators); however, no new data will be collected. Withdrawing from the study will not have any consequences for the participant. Participants’ identities will not be revealed in the publication or presentation of any results from this study.

### Access to Data

The data sets generated during and/or analyzed during this study are not currently publicly available due to the ongoing trial but will be available from the corresponding author upon reasonable request.

### Dissemination Policy

The results of this study will be disseminated through publications and presentations at the scientific conferences.

## Results

This study is currently ongoing and actively recruiting participants. So far, 5 participants have completed the trial, 1 is currently in active treatment, and 8 participants are on the waitlist to be screened. We anticipate initial results being available in the fall of 2023. This proposal was presented as a poster presentation at the Research to Reality Global Summit on Psychedelic-Assisted Therapies and Medicine, held in May 2022 in Toronto, Canada.

## Discussion

### Expected Findings

Developing effective interventions for acute suicidality in high-risk populations such as BD remains a major therapeutic challenge. Ketamine, an NMDA receptor antagonist, represents a promising intervention due to its rapid antidepressant and antisuicidal effects [11]. However, our understanding of its underlying neurophysiological mechanisms for suicidality in BD is limited. TMS-EMG and TMS-EEG can be used to produce a reliable neurophysiological measure of NMDA receptor–mediated neurotransmission [15]. To our knowledge, this is the first study to investigate the safety/feasibility and neurophysiological changes of repeated 40-minute ketamine infusions to treat suicidality in patients with BD. Results from this study will (1) inform the development of a larger, adequately powered randomized controlled trial to test the feasibility of a series of IV ketamine infusions to treat suicidality in patients with BD and (2) determine potential neurophysiological markers of clinical response.

### Limitations

In general, a lack of a control group and a small sample size are limitations in ketamine clinical trials. However, given that this is a pilot open-label clinical trial, the sample size is sufficient (ie, the goal is not to assess generalizability or statistical significance). The lack of blinding in the control group is a common concern in ketamine trials because a placebo with comparable effects does not exist, and it is impossible to ensure blinding to the full extent. Because the study is conducted at a single site of a large psychiatric academic facility, the results may not be fully transferable to a broader population; however, the study will provide preliminary data on feasibility and safety to be used in a larger, adequately powered and controlled trial. Moreover, a small sample size may not allow drawing any meaningful conclusions around neurophysiological markers of response; however, results will inform a further larger clinical trial where we can test neurophysiological markers more accurately.

### Clinical Significance

BD is an incapacitating psychiatric condition with high mortality rates due to suicide, where 25%-60% of patients will attempt suicide at least once in their lifetime, and about 10%-15% die by suicide. Current treatment options for suicidality are very limited. Subanesthetic doses of ketamine have been shown to have rapid antisuicidal effects [5]; however, research exploring ketamine’s biological targets of treatment response remains limited. This study will provide preliminary data on the feasibility of repeated IV ketamine infusions to treat suicidality in patients with BD and investigate potential neurophysiological mechanisms to identify the biomarkers of clinical response.

## Appendix

Multimedia Appendix 1 SPIRIT (Standard Protocol Items: Recommendations for Interventional Trials) flowchart.

Multimedia Appendix 2 SPIRIT (Standard Protocol Items: Recommendations for Interventional Trials) checklist.

Multimedia Appendix 3 Participant consent form.

## Abbreviations

ASKP: American Society for Ketamine Physicians
BD: bipolar depression
CAMH: Centre for Addiction and Mental Health
CLEARR: Clinical Engagement and Research Recruitment
CNS: central nervous system
DEEPP: Anti-suicidal Response to Ketamine in Treatment-Resistant Bipolar Depression
DSM-V: Diagnostic and Statistical Manual of Mental Disorders, 5th edition
EEG: electroencephalography
EMG: electromyography
FDA21 CFR: US Food and Drug Administration Code of Federal Regulations Title 21
GABA: gamma-aminobutyric acid
HIPAA: Health Insurance Portability and Accountability Act
HRSD-24: 24-item Hamilton Rating Scale for Depression
IV: intravenous
MINI: Mini-International Neuropsychiatric Interview
NMDA: N-methyl-d-aspartate
PI: principal investigator
PIPEDA: Personal Information Protection and Electronic Documents Act
REDCap: Research Electronic Data Capture
SPIRIT: Standard Protocol Items: Recommendations for Interventional Trials
SSI: Scale for Suicide Ideation
TMS: transcranial magnetic stimulation
WHODAS 2.0: World Health Organization Disability Assessment Schedule
YMRS: Young Mania Rating Scale
