# Supplementary material for: Neural Correlates of the DEEPP (Anti-suicidal Response to Ketamine in Treatment-Resistant Bipolar Depression) Study: Protocol for a Pilot, Open-Label Clinical Trial
Source: JMIR Res Protoc. 2023 Jan 27;12:e41013. doi: 10.2196/41013 (PMC9919457; doi:10.2196/41013)
Supplement: Multimedia Appendix 1 [file resprot_v12i1e41013_app1.docx]

# Appendix 1: SPIRIT Flow Chart Study Visits

# Neural correlates of anti-suiciDal rEsponse to kEtamine in treatment-resistant biPolar depression (DEEPP-Study): Protocol for a Pilot, Open-Label Clinical Trial

| Visit | Intervi ew and Questi on- naires | TMS- EMG/ EEG | Treatment  (Ketamine IV) | Follow-up with physician | Time |
| --- | --- | --- | --- | --- | --- |
| Pre-treatment Phase (Screening and Baseline Visits) | | | | | |
| Screening Visit*  **This visit can occur on one day or split over 2 separate days* | X |  |  |  | 2-2.5  hours |
| Baseline Visit | X | X |  |  | 1.5-2  hours |
| Treatment Phase (Week 1) | | | | | |
| Treatment Visit 1* |  | X | X |  | 3 hours |
| Treatment Visits 2* |  |  | X |  | 2 hours |
| Post treatment session # 2** | X |  |  | X | 1 hour |
| Treatment Phase (Week 2) | | | | | |

| Treatment Visits 3* |  |  | X |  | 2 hours |  |
| --- | --- | --- | --- | --- | --- | --- |
| Treatment Visits 4* |  |  | X |  | 2 hours |  |
| Post treatment session # 4** | X |  |  | X | 1 hour |  |
| Treatment Phase (Week 3) | | | | | |  |
| Treatment Visit 5* |  |  | X |  |  |  |
| Treatment Visit 6* |  |  | X |  |  |  |
| Post treatment session # 6** | X |  |  | X | 1 hour |  |
| Treatment Phase (Week 4) | | | | | |  |
| Treatment Visit 7* |  |  | X |  |  |  |
| Treatment Visit 8* |  |  | X |  |  |  |
| Post-treatment Phase | | | | | |  |
| Post-treatment/ Discontinuation Monitoring  Visit (within one week after last ketamine treatment; within one month of the last  study visit in case of discontinuation) | X | X |  | X | 1.5-2  hours |  |
| Follow up assessment  (within one month after last ketamine treatment) | X |  |  |  | 1 hour |  |
