# Supplementary material for: Neural Correlates of the DEEPP (Anti-suicidal Response to Ketamine in Treatment-Resistant Bipolar Depression) Study: Protocol for a Pilot, Open-Label Clinical Trial
Source: JMIR Res Protoc. 2023 Jan 27;12:e41013. doi: 10.2196/41013 (PMC9919457; doi:10.2196/41013)
Supplement: Multimedia Appendix 3 [file resprot_v12i1e41013_app3.pdf]

camh

CAMH INFORMED CONSENT FORM

Study Title: Neural correlates of anti-suiciDal rEsponse to kEtamine in treatment resistant biPolar dePression (DEEPP-Study). Pilot Project

Principal Investigator: Yuliya Knyahnytska, MD, PhD, (416) 535-8501 ext. 34648

Co-Investigators: Daniel Blumberger, MD, MS; FRCPC
Daphne Voineskos, MD, PhD, FRCPC
Tyler Kaster, MD, FRCP
Alisson Trevizol, MD, FRCPC
Reza Zomorrodi, PhD, Project Scientist

Funder(s): Discovery Seed Fund

INTRODUCTION

This consent form describes a research study and what it means to take a part in it. This form may use words you do not understand. Please ask the research team any questions you have and to explain anything that you do not understand.

It is your choice whether you decide to take part in this study or not. You do not have to take part if you don't want to. If you do decide to participate, you can change your mind later. No matter what you decide, it will not affect the care or any other services that you receive at CAMH.

Please take as much time as you need to decide. If you'd like to, you can talk about this study with other people (for example, your family, friends, your family doctor, and other health professionals).

There is a section at the end of this document that explains how the study may be conducted remotely when extra precautions are required and how some of the procedures may differ.

WHY IS THIS STUDY BEING DONE?

Suicide is common and the rates are progressively rising. Bipolar disorder (BD) is a condition that causes unusual shifts in mood, energy, activity and concentration. Interventions such as medication, cognitive behavioral therapy (CBT) and electroconvulsive therapy (ECT) have demonstrated anti-suicidal effects. However, some of these regimens are often difficult to tolerate and may take months to take effect.

Ketamine, a widely used and approved anesthetic agent, when used in sub-anesthetic doses has been shown to rapidly improve depressive symptoms. Studies have also shown it has anti-suicidal

effects. It can be administered in a variety of ways, with intravenous (IV), and intranasal (IN) most commonly used. IV ketamine administration has been shown to be most helpful. This study will examine the safety, tolerability and effectiveness of IV ketamine in those with BD experiencing depressive symptoms and suicidal thoughts. We also will look into brain markers of treatment response so we can predict better in the future who may benefit from this treatment the most.

The use of *Intravenous ketamine* in this study is investigational. It is being used in this study in a different way than how it is normally used, and Health Canada has not approved this use outside of a research study. Health Canada is the regulatory (government) body that oversees the use of natural health products, drugs and medical devices in Canada; and this study has received an approval from Health Canada.

About 30 people will participate in the study. They will visit the Centre for Addiction and Mental Health (CAMH).

WHAT WILL HAPPEN DURING THIS STUDY?

Research studies have strict requirements on who can join to make it safer for participants while ensuring study results are reliable. You will complete some tests and procedures to make sure that you meet the specific requirements of this study. You may find out that you are not eligible to take part. If the research team learns something about your health that is important to your medical care, they will let you know.

In order to participate in this study, we will ask you to do:

- clinical questionnaires
- brain activity and inhibition testing, such as TMS-EMG, and EEG (details below)

If eligible you will be enrolled in a study.

STUDY VISITS AND PROCEDURES

Screening and Baseline Visit: The first study visit will be a screening visit. This visit will involve an interview to assess your eligibility and to confirm that you can safely undergo treatment. The results of the tests/questions at the screening visit help the researchers to decide whether you can continue in this study. The results of all tests and interviews are completely confidential. In addition, you will complete routine tests such as bloodwork, ECG, and physical assessment as part of the screening process. Routine blood work and ECG will be ordered and completed as per standard clinical practice. It is possible that you may have already completed some of these tests such as bloodwork or ECG prior to study entry as part of your clinical care. The results will be available for screening purposes and will be reviewed by the study doctor prior to treatment start. This visit should take approximately 2 hours. Following eligibility confirmation, you will also complete some baseline measures, which include questionnaires and a neurophysiology session. The baseline visit will last approximately 1.5 hours.

Treatment Visits 1-8: The treatments will take place over a 4-week time frame, two days a week for a total of eight treatment sessions. The duration of your visits will vary between 2 hours to 3 hours. It is required that you have an escort to take you home after each treatment visit. An escort may be a relative, friend, neighbour, case worker, etc. A taxi driver is not considered a suitable escort.

camh

If you miss more than 2 consecutive treatment sessions, your treatment as part of the study will be stopped, as missing treatment sessions compromises the efficacy of the treatment.

Monitoring Visits: The study team will follow your progress with additional monitoring visits after every two treatments. It should take around 45-60 minutes to complete this monitoring visit. A follow-up visit will be scheduled 1 month after the end of your treatment course.

Brain Activity and Inhibition Testing (TMS-EMG/EEG):
Transcranial magnetic stimulation (TMS) is a method used to measure brain inhibition. TMS excites nerves over the area of the brain involved in moving your hand muscles. When the nerves are stimulated, this causes the muscles in your hand to move, which will be recorded and later analyzed. Brain activity and inhibition during TMS will be assessed using electroencephalography (EEG) and electromyography (EMG).

- You will be seated in a comfortable chair and we will attach soft foam electrodes to the skin surface over your hand muscles; these electrodes will then be connected to a recorder that will record the activity of your hand muscles.
- It takes approximately 30 minutes to put on the EEG cap and get it ready for recording. The cap contains many recorders that record your brain activity. There is gel on the inside of the cap that may be sticky; you will be allowed to rinse it out after the test.
- A magnetic coil will be held on the surface of your scalp. When the magnetic stimulation is applied, you will feel a twitch or small movement in your hand, but there should be no pain.
- The TMS measures of brain physiology will be taken from your motor cortex (the part of the brain that controls movement) and the prefrontal cortex (the part of the brain that controls thinking).

There will be one scheduled before your first treatment session, one after your first treatment and one after your treatment course is complete, each lasting around 1 – 1.5 hours.

<u>INTERVENTION</u>:

Ketamine will be given across eight sessions, twice a week for 4 weeks. Each session will take about 2-2.5 hours. We will use ketamine hydrochloride solution which will be provided by CAMH pharmacy. A qualified medical personnel will be administering the Ketamine intravenously. The dosage will be determined by a study doctor based on physical and clinical parameters and will range between (0.5-0.8 mg/kg).

For the treatment session you will be staying in a separate dedicated space, and will be provided with ear plugs or headphone to minimize exposure to light and sound during treatment time. In order to minimize risk of side effects, we ask that you:

- Avoid eating large meals at least 2 hours before administration
- Avoid drinking coffee at least 30 minutes prior to administration
- Avoid use alcohol, substances, hypnotics, and anxiolytics for at least 24 hours prior to your treatment session.

You will need to stay on-site for 2 hours after ketamine is given to ensure we can monitor short-term side effects from ketamine use. Your vital signs will be monitored every 30 minutes. This is for your safety.

After two hours, you may still be under the influence of ketamine, even if you do not feel like you are. You will need to make arrangements before your visit for someone to escort you home. We will ask you to confirm this arrangement before starting each of your treatment sessions.
You will not be allowed to drive or leave CAMH premises on your own after the procedure. If no escort is available to accompany you home after the treatment, you will not be able to receive your treatment due to potential safety concerns. You should also avoid making important decisions or operate machinery (including driving) 24-hr post ketamine administration. Recommendations about arranged escort are in line with those you have had during your electroconvulsive therapy (ECT) course.

After you have completed study visits, you will have a final assessment which will include TMS-EMG/ EEG and questionnaires, and may take up to 45-60 min to complete. You may schedule a follow up with the study physician at the end of the trial to discuss your discharge planning.

Calendar of Visits

Boxes marked with an X show what will happen at each visit:

**Visit**	Interview and Questionnaires	TMS-EMG/ EEG	Treatment (Ketamine IV)	Follow-up with physician	Time
Pre-treatment Phase (Screening and Baseline Visits)					
Screening Visit* *This visit can occur on one day or split over 2 separate days*	X				2-2.5 hours
Baseline Visit	X	X			1.5-2 hours
Treatment Phase (Week 1)					
Treatment Visit 1*		X	X		3 hours
Treatment Visits 2*			X		2 hours
Post treatment session # 2**	X			X	1 hour
Treatment Phase (Week 2)					

Treatment Visits 3*			X		2 hours
Treatment Visits 4*			X		2 hours
Post treatment session # 4**	X			X	1 hour
Treatment Phase (Week 3)					
Treatment Visit 5*			X		
Treatment Visit 6*			X		
Post treatment session # 6**	X			X	1 hour
Treatment Phase (Week 4)					
Treatment Visit 7*			X		
Treatment Visit 8*			X		
Post-treatment Phase					
Post-treatment/ Discontinuation Monitoring Visit (within one week after last ketamine treatment; within one month of the last study visit in case of discontinuation)	X	X		X	1.5-2 hours
Follow up assessment (within one month after last ketamine treatment)	X				1 hour

*Treatment visits will occur on non-consecutive days
** Post-treatment assessments can be completed the following day after treatment or any time prior to the next treatment visit on the following week.

WHAT ARE THE RISKS, HARMS OR DISCOMFORTS?

You may experience some physical and psychological changes during ketamine sessions. There is a two-hour monitoring period associated with ketamine administration to make sure that if you experience any changes you return to your normal before you leave. Risks described below are rare, and there is no expected frequency of the risks.

Potential side effects may include:
- Fatigue
- Dizziness
- Passing out

- Anxiety
- Hallucinations
- Panic attacks
- Irritability
- Mood changes
- Risk of Dependency*
- Severe headache
- Vision changes
- Chest pain
- Shortness of breath
- Confusion
- Memory impairment
- Increase in need to urinate, or painful urination
- Anaphylaxis
- Transient redness
- Rash

Risk of Dependency: Ketamine is classified as a controlled substance due to its potential for abuse and addiction. It can be abused in a number of ways, including via injection, snorting, or orally. Ketamine can produce vivid dreams and a feeling that the mind is separated from the body. This effect, called “dissociation,” is also produced by the related drug. Those who use on regular basis may soon become tolerant to the dissociative effects of the drug, meaning some may continue to escalate doses. Some people may develop dependency, and continue to use ketamine even when they plan not to or despite its negative effects. If you are someone who regularly uses substances, you may be at higher risk of developing dependency.

In this study, ketamine is dispensed by the pharmacy and given to you by a trained physician with close monitoring. Doses are individually calculated, and treatment sessions are structured to prevent tolerance building. If you use ketamine outside of the trial without monitoring, you maybe at a higher risk of developing an addiction and variety of side effects. We would very strongly discourage you from using ketamine outside of the context of this trial. If you have more questions, please discuss with your medical provider or study doctor.

TMS Risks: When the stimulation (TMS) is applied to your head, you will hear a short click from the machine. You will be given ear-insert headphones, and white noise will be played to mute the sound. At certain positions on the head, the stimulation may cause eyes to blink or a brief contraction of the scalp, neck, trunk or upper arm muscles. You may find these contractions annoying, but they should not be painful. Some people may experience mild headache or shoulder stiffness after testing but these symptoms will usually go away in 24 hours. Typically, acetaminophen is enough to get rid of these symptoms. If you have further concerns you may contact the investigators at any time. Magnetic brain stimulation has been used on thousands of individuals in the United States, Canada and Europe over several years without any serious problems.

ARE THERE REPRODUCTIVE RISKS?

Researchers do not know what effects short exposure to IV ketamine might have on reproduction and an unborn baby. However, to mitigate potential side effects, you will not be allowed to participate in the study if you may be pregnant or breastfeeding. We expect you to use a medically acceptable form of birth control method during the trial. Such methods include hormonal (e.g. birth control pills, patch, hormone injections or implants), IUD, double barrier methods (e.g. male condom with diaphragm, male condom with cervical cap) or abstinence if that is your preferred and usual lifestyle.

If you do become pregnant or father a child during the study, please tell the research team right away.

WHAT ARE MY RESPONSIBILITIES AS A STUDY PARTICIPANT?

Before each ketamine session, you must arrange for a ride home with a companion.

If you choose to participate in this study, we ask you to tell us about:

- any uncontrolled current medical conditions which may impact your participation.
- all prescription and non-prescription medications and supplements, including vitamins
- and herbal supplements, and check with the research team before starting, stopping or changing any of these. This is for your safety as these may interact with the intervention you receive in this study.
- if you are thinking about participating in another research study, or if you change your mind about participating in this study.
- if anything about your health has changed, including if you become pregnant or father a child while participating on this study.
- If you have any questions or anything is worrying you about the study.

HOW LONG WILL I BE IN THE STUDY?

The study intervention will last for about four weeks. The study also includes baseline and post assessments which will be done during different weeks. Additionally, a follow-up visit is scheduled one month after the end of your treatment course. Therefore, in total you will be involved in this study for a total of 8-10 weeks.

This study should take about two years to complete participant recruitment and the results should be known in about three years' timeframe.

WHAT OTHER CHOICES MIGHT I HAVE?

You do not have to take part in this study to receive treatment or care at CAMH. If you decide not to take part in the study, you can still continue with other services available from your most

responsible care provider and/or CAMH programs if you qualify.

Other treatment options may include, but are not limited to

- Medication recommendations
- Referral to the intensive day treatment program(s) if qualified

Please talk to your doctor or the research team about the benefits and risks of the other options and whether they may be suitable for you before you decide to take part in this study.

CAN I LEAVE THE STUDY?

You can change your mind at any time and decide to not take part anymore (called withdrawal). The research team may ask why you are withdrawing for reporting purposes, but you do not need to give a reason if you do not want to. If you decide to stop, you may also be asked questions about your experience with the study intervention and to undergo tests to check your health. If you decide to leave the study, please contact the research team to let them know.

If you withdraw from the study, information that was recorded before you withdrew including the results of tests done will be kept by the researchers (it will not be destroyed), but no more information about you will be collected, sent to, or shared with anyone else after you withdraw your permission.

CAN MY PARTICIPATION END EARLY?

The researchers may take you out of the study early if:

- You are experiencing side effects, and the study physician determines it is not in your best interest to continue with the study
- You are unable to complete all required study procedures
- You decide to withdraw from the study for whatever reason
- The study is stopped early or cancelled
- If you plan to or become pregnant
- There may be other reasons to take you out of the study that the research team does not know at this time

If this happens, it may mean that you would not receive the study intervention for the full period described in this consent form.

If you are removed from this study, the research team will discuss the reasons with you and plans will be made for your continued care outside of the study.

WHAT ARE THE BENEFITS?

The researchers do not know whether or not you will benefit from taking part in the study.

Your participation can also be very helpful in contributing to the science so we can better understand how to treat suicidality & ideation. Researchers hope the information learned from

this study will benefit other people with suicidality in depression in the future.

PRIVACY AND CONFIDENTIALITY

What personal information will be collected?

If you decide to participate in this study, the research team will collect personal information or personal health information for the purposes of the study. Information will be collected directly from you (for example, during interviews and on questionnaires) and from your medical records. To determine if you meet the requirements to participate in this study, a member of the research team will need to access your CAMH Health Records.

Personal information is information of a personal nature about you that could identify you. Personal health information is information about your physical or mental health or the health care that you receive that could identify you. Information collected for this study may identify you when used alone (e.g. your name) or when combined with other available information about you.

The personal information or personal health information that may be collected, used, and stored in connection with this study will include:

- Demographic information: name, address, phone number, email address, medical record number, OHIP (health card) number, date of birth; sex and gender; race/ethnicity, and a

- name of your treating physician
- specific diagnostic tests *(e.g., blood samples, ECG)*
- information about your health including the dates and results of medical tests or procedures if applicable
- results of study-specific assessments and tests

How will my personal information be kept confidential?

Directly identifying information (e.g. name, DOB, gender, etc.) will be removed from the rest of the information the research team collects about you and replaced with a code ('coded data'). The research team will have a list that links your name to your code so that your coded data can be linked back to you if necessary. This list will be kept separate from the coded data in a secure place. Even though the likelihood that someone may identify you from the coded data is very small, the risk can never be completely eliminated.

When the results of this study are published, your identity will remain confidential. It is expected that the information collected during this study will be published/ presented to the scientific community at meetings and in journals. This information may also be used as part of a submission to regulatory authorities around the world to support the approval of the study intervention.

Limitations to Confidentiality

There are some special circumstances in which the researchers may need to share information that you provide and in which your confidentiality may not be protected. Examples of these

special circumstances are: (1) members of the research team have reasonable grounds to believe that disclosing information is necessary to eliminate or reduce a significant risk of bodily harm to yourself or others; (2) if there is reasonable suspicion that a child is at risk of harm or neglect or is witnessing parental violence; (3) if a healthcare professional has engaged in sexual behavior with a patient, (4) if our files are subpoenaed by a court of law or where public health laws require that health professionals report a communicable disease.

What information will be added to my medical records?
Your participation in this study will also be recorded in your medical record at CAMH. This is for clinical safety purposes. If you participate in this study, information about you from this research project may be stored in your hospital file and in the hospital computer system. CAMH shares patient information stored in our electronic health record with other hospitals and healthcare providers in Ontario so they can access the information if it is needed for your clinical care. The study team can tell you what information about you will be stored electronically and may be shared outside of CAMH. If you have concerns about this, or have any questions, please contact the Information and Privacy Office at 416-535-8501 x33314 or by email at privacy@camh.ca

Who may have access to my medical and/or study records?

Members of the research team and authorized representatives of CAMH, including the CAMH

Research Ethics Board and Quality Assurance team, will have access to your medical and/or study records for use in connection with this study.

For your safety, authorized personnel from CAMH will confirm the name and date of birth that you provide with your government issued identification.

People from the following organizations may come to CAMH to look at your medical and study records (including personal information and personal health information) to check that the information collected for the study is correct and follows proper laws and guidelines:

- Health Canada (because they oversee the use of drugs in Canada)

Coded data may also be sent to the people and organizations listed above.

What else should I know about privacy and confidentiality?

Our research team may occasionally communicate with you by using emails (e.g. trying to reach you if you do not respond to calls, send appointment info), or you may communicate to research team through email with questions or concerns. We want you to be aware that the security of information sent by e-mail cannot be guaranteed. We ask you not to use personal sensitive information by e-mail. Please do not use e-mail to communicate emergency or urgent health matters – please contact your clinician or family doctor. If it is a medical emergency, call 911. Please let the research team know if you do not want to be contacted by e-mail.

With your permission, some basic information (e.g. demographics, medications, safety screening, brain stimulation history, etc.) gathered as part of the screening process will be stored in a centralized electronic database and may be shared with other research personnel affiliated with the Temerty Centre. This data will be used to safely track your participation and to

better match you with current and future studies that you may be eligible for if you consent to be re-contacted. Only investigators/research teams affiliated with the Temerty Centre will have access to this secured database, and will adhere to all appropriate measures to safeguard the confidentiality of your information. Only data collected from participants at CAMH will be included in this database.

WILL MY FAMILY DOCTORS/HEALTH CARE PROVIDERS KNOW I AM PARTICIPATING IN THIS STUDY?

Your most responsible physician (MRP) will receive a consultation note from the study doctor; they may also receive discharge note after you complete this trial. If you do not want them to be informed, please discuss this with the research team ahead of time.

WILL INFORMATION BE AVAILABLE ONLINE?

A description of this clinical trial will be available on *clinicaltrial.gov* website. This website will not include information that can identify you. You can search this website at any time.

IS THERE A COST TO ME?

Taking part in this study may result in added costs to you. For example:
- There may be costs associated with study visits. For example, parking or transportation, child care, or snacks/meals during your stay.
- You may miss work as a result of participation in this study.

The research team will do their best to minimize these extra costs by arranging your research visits around your clinical appointments where possible.

WILL I BE ABLE TO CONTINUE WITH THE INTERVENTION AFTER THE STUDY IS OVER?

You may not be able to continue with the study intervention after your participation in the study has ended. This may be because:
- The intervention is currently only available to research participants
- The intervention may not turn out to be effective or safe
- The intervention may not be approved for use in Canada
- Your health care providers may not feel it is the best option for you
- You may decide it is too expensive and insurance coverage may not be available
- The intervention, even if approved in Canada, may not be available free of charge

The research team will talk to you about your options.

WILL I BE COMPENSATED?

You will not receive any monetary compensation for taking part in this study.

camh

WHAT HAPPENS IF I AM INJURED IN THE STUDY?

If you get injured or have side effects from participation in this study, medical care will be provided in the same way you would normally get medical care (e.g. study doctor attending; visit ED, or communicating back to your treating physician*).* The costs of your medical treatment will be paid for by the provincial medical plan or by seeking reimbursement from your private medical insurer (if any) to the extent that such coverage is available.

There may be extra costs that are not covered by provincial insurance or your private medical plan. Examples of these extra costs could be medications or treatments to treat side effects that you may experience. If you have private health care insurance, the insurer may not pay for these added costs.

WHAT ARE MY RIGHTS AS A RESEARCH PARTICIPANT?

If the researchers learn about new information that may be important to your decision to stay in the study they will tell you about it in a timely manner.

You can find out the results of this study once the entire study is completed. Please contact the research team to learn the results or let the research team know if you would like to learn the results. The results of this study will be available on the clinical trial registry.

By signing this form you do not give up any of your legal rights against the study doctor, sponsor or involved institutions for compensation, nor does this form relieve the study doctor, sponsor or their agents of their legal and professional responsibilities.

You will be given a copy of this signed and dated consent form.

WHAT IF RESEARCHERS DISCOVER SOMETHING ABOUT ME?

During the study, the researchers may learn something about you that they did not expect. For example, the researchers may find out that you have another medical condition.
If any new clinically important information about your health is obtained as a result of your participation in this study, you will be given the opportunity to decide whether you wish to be made aware of that information.

WHO DO I CONTACT FOR QUESTIONS?

If you have questions about this study, or if you experience a research-related injury, you can talk to the Researcher who is in charge of the study at CAMH. That person is:

Dr. Yuliya, Knyahnytska, Principal Investigator	416-535-8501, Ext. 34648
Name	Telephone

If you have questions about your rights as a participant or about ethical issues related to this study, you can talk to someone who is not involved in the study at all. That person is the Chair of the Research Ethics Board (REB). The REB is a group of people responsible for the ethical oversight of this study. The Chair of the REB can be reached by telephone at 416-535-8501 ext. 34020.

WHO DO I CONTACT IN THE CASE OF AN EMERGENCY?

In case of an emergency, please have a clinician contact CAMH switchboard at (416) 535-8501 and ask them to page the study doctor for the Ketamine IV DEEPP Trial.

Modified Procedures

All study visits, except for treatment sessions and TMS-EMG/ EEG sessions, will be conducted virtually using videocoference software (Webex), or the telephone if you are not able to use the videoconference software. Videoconferencing is preferred, as some of our assessments require behavioral observation. If you have no access to a device with videoconferencing, assessments can also be completed onsite during your in-person visit. You will be provided with a private room and a device with videoconferencing capabilities to complete the assessment at CAMH. Like online shopping, videoconferencing technology has some privacy and security risks. It is possible that information could be intercepted by unauthorized people (hacked) or otherwise shared by accident. This risk can't be completely eliminated, however CAMH has approved the use of WebEx for videoconferencing sessions because the appointments take place over a secure encrypted network. The research team will confirm your identity at the beginning of the call and may also ask to see a piece of government-issued ID, via video, during the session.

Video sessions can be conducted using your cell phone, tablet or personal computer enabled with a camera/microphone and internet connection. You should use your home computer or personal device, and not a shared or work device. To use WebEx, an e-mail will be sent to you including the instructions for how to log-in. Self-report questionnaires will be completed using screen share, or verbally completed over Webex or telephone.

The security of information sent by e-mail cannot be guaranteed. Please do not communicate personal sensitive information by e-mail. E-mail is not routinely monitored outside of work hours. Please do not use e-mail to communicate emergency or urgent health matters – please contact your clinician or family doctor. If it is a medical emergency, call 911. By signing this form, you agree that the research team may contact you by email for the purposes of this study.

For your safety, the research team will ask you for an emergency contact number, alternate phone number and your address before they start the call. They may follow-up with you after the session if you leave early. If at any time, we are concerned for your safety, we may contact you, your emergency contact or emergency responders to follow-up.

For these videoconference sessions, please try to find a quiet place where you can be by yourself and will not be disturbed and use earphones if you can. It's a good idea to test out the system a few minutes before the session to make sure the connection and sound are working. You or the research team can stop the session at any time, including if there are technical difficulties. If there are technical issues, one of our technical staff may join the call to provide support.

Treatment sessions will be conducted in person. You will be told ahead of time what time you should arrive for your treatment in order to minimize the number of people in the waiting room. Prior to entering CAMH each day, every person is screened for COVID-19 symptoms and contacts. Any person who is found to have symptoms or contacts as per the screening protocol

(known as a positive screen) will not be permitted to enter CAMH. If you are found to be a positive screen on any given day during the study, treatment will not be allowed to continue that day. Depending on the determination of the Infection Prevention and Control team at CAMH, we may or may not be able to resume treatments. If you miss more than 2 scheduled treatments due to a positive screen it will be at the discretion of the treatment team whether or not you can continue to receive treatment. If you do not have COVID-19 symptoms or contacts as per the screening protocol (known as a negative screen), you will be given a wristband to indicate you have been screened for that day. A CAMH staff will then escort you to the waiting room. Once you have recovered from the treatment the CAMH staff will take you to the person who is escorting you home.

During the brain physiology sessions, you will be required to wear a mask. The research staff will be wearing a mask, face shield and gloves. The research staff will maintain a physical distance whenever possible but there will be extended periods where they will be within 6ft/2m. All of the equipment and furniture that they use will be disinfected before and after the session. You will not be allowed to rinse out your hair at the end of the session, but we will provide you with a cap for your commute home.

Study Title: Neural correlates of anti-suiciDal rEsponse to kEtamine in treatment resistant biPolar dePression (DEEPP-Study). Pilot Project

SIGNATURES

- All of my questions have been answered,
- I have read each page and I understand the information within this informed consent form,
- I allow access to my personal health information, medical record and research data as explained in this consent form,
- I do not give up any of my legal rights by signing this consent form,
- I understand that my family doctor/health care provider will/may be informed of my participation in this study
- I agree, or agree to allow the person I am responsible for, to take part in this study.

Should I be interested in future research studies, I agree to be contacted in the future.

☐ Yes, I agree

☐ No, I do not agree

Signature of Participant	PRINTED NAME	Date
Signature of Person Conducting the Consent Discussion	PRINTED NAME	Date
